# The Effect of Implementing Orem's Self‐Care Model on Balance and Fatigue in Patients With Diabetic Neuropathy: A Clinical Trial

**DOI:** 10.1002/nop2.70985

**Published:** 2026-10-30

**Authors:** Atefeh Sadeghi, Mohammad Malekzadeh, Abolfazl Dehbanizadeh, Seyed Javad Sadat

**Affiliations:** ^1^ Student Research Committee Yasuj University of Medical Sciences Yasuj Iran; ^2^ Health Research Centre Yasuj University of Medical Sciences Yasuj Iran; ^3^ Department of Medical–Surgical Nursing, School of Nursing Yasuj University of Medical Sciences Yasuj Iran

**Keywords:** balance, diabetic neuropathy, fatigue, nursing, Orem's self‐care model

## Abstract

**Aim:**

This study aims to determine the effect of applying Orem's self‐care model on balance and fatigue in patients with diabetic neuropathy.

**Design:**

Randomized clinical trial.

**Methods:**

Seventy diabetic neuropathy patients referred to Shahid Mofateh Clinic, affiliated with Yasuj University of Medical Sciences (YUMS), were chosen for this clinical trial study via convenience sampling and randomly assigned to an intervention group (*n* = 35) or a control group (*n* = 35). The intervention group received care based on Orem's self‐care model from a graduate nursing student over 2 months, followed by 1 month of self‐care at home. The control group received no intervention. Both groups completed the research questionnaires (the Multidimensional Fatigue Inventory and the Berg Balance Test) at pretest and posttest. Data were analysed in SPSS version 27 using an ANCOVA test.

**Results:**

Before the intervention, there were no significant between‐group differences (*p* > 0.05). After the intervention, fatigue was significantly lower in the intervention group, with an adjusted mean difference of −9.40 points (95% CI: −11.09 to −7.71; *p* < 0.001; partial *η* ^2^ = 0.647). Balance scores were significantly higher in the intervention group across low, mean, and high baseline levels, with adjusted mean differences of 7.01, 5.77, and 4.52 points, respectively (all *p* < 0.001). The group × baseline balance interaction was significant (F(1,66) = 5.69, *p* = 0.020; partial *η* ^2^ = 0.079).

**Implications for Nursing:**

Nurses are recommended to apply Orem's self‐care model as an effective structured intervention to reduce fatigue and improve balance in patients with diabetic neuropathy.

**Patient or Public Contribution:**

Patients with diabetic peripheral neuropathy (diagnosed by an internist using TCSS) who participated in this randomized clinical trial evaluating Orem's self‐care model effects on balance and fatigue.

## 1 Introduction

Diabetes is a complicated, long‐term metabolic condition that imposes a substantial burden on healthcare systems. According to a report by the International Diabetes Federation (IDF), 783 million people worldwide are expected to have diabetes by 2045 (International Diabetes Federation 2021). In Iran, 14.15% of people over 25 had diabetes in 2021, and if effective prevention is not implemented, this number is expected to rise to 9.2 million by 2030 (Moradpour et al. 2022). Peripheral neuropathy, blindness, kidney failure, cardiovascular problems, death, and amputation are all primarily caused by this disease (Perveen et al. 2024). The most frequent microvascular consequence in diabetic individuals is sensory–motor neuropathy (Amorim et al. 2019). Peripheral neuropathy refers to a broad spectrum of tissue damage resulting in dysfunction of the peripheral nervous system (Wang et al. 2018). Approximately half of diabetic patients experience diabetic peripheral neuropathy (DPN) during their lifetime, which can impair proprioception and consequently compromise balance (Feldman et al. 2019; Schmidt 2018). Furthermore, individuals with DPN are up to 15 times more likely to report injuries and experience reduced safety during standing and walking compared with healthy individuals, increasing their risk of falls and fall‐related injuries (Zhao et al. 2021; Alissa et al. 2024). Painful DPN is also common, with approximately 25.2% of individuals with DPN experiencing painful neuropathy (Elliott et al. 2024). Owing to sensory and motor impairments associated with DPN, including lower‐limb weakness and impaired balance, patients may experience reduced mobility and functional limitations (Alissa et al. 2024; Nakashima et al. 2025). Moreover, muscle weakness and reduced physical functioning associated with DPN may contribute to fatigue and decreased energy levels (Nakashima et al. 2025). Fatigue is a common and clinically significant symptom among individuals with diabetes, affecting approximately 50% of those with type 2 diabetes (Romadlon et al. 2022). Fatigue has multiple dimensions and includes physical and cognitive fatigue. Cognitive fatigue can negatively affect cognitive tasks and social duties. Psychological factors, including emotional distress or depression, may also be linked to fatigue (Sienko et al. 2018). Controlling type 2 diabetes and its complications is challenging because combination therapies are more effective than medication alone, which often fails to achieve the intended therapeutic effects (Feldman et al. 2019). Furthermore, research indicates that self‐care is a deliberate practice essential for patients' ongoing treatment (Sari et al. 2020).

The effectiveness of diabetes treatment largely depends on the patient's self‐care, as the patient is responsible for more than 95% of diabetes care. Self‐care is an active, practical process through which individuals maintain and improve their health, prevent and manage disease, and reduce both short‐ and long‐term complications (Fereidooni et al. 2024). A study by Sarabi et al. (2024) revealed that 93% of diabetic patients had low awareness, and 93.3% had low self‐care performance scores. (Sarabi et al. 2024). According to findings from other studies, 68.5% of diabetic patients have inadequate self‐care abilities, and only 16.2% of them follow self‐care practices (Zeidi et al. 2021). According to Kiçaj et al. (2024), several educational approaches can improve self‐care ability; however, patients need sufficient knowledge and skills to understand their self‐care needs and take appropriate actions to enhance their self‐care capacity (Kiçaj et al. 2024). Self‐care actions that lead to the prevention of complications and improvement of quality of life in these patients include food selection, physical activity, foot care, and blood sugar monitoring, which are necessary for patient empowerment. Orem's self‐care theory is a nursing theory that is useful in this context (Nahid et al. 2022). Orem's self‐care model is one of the most useful models because of the various demands that patients have for self‐care and the necessity for healthcare professionals, particularly nurses, to assess the degree of self‐care and create a comprehensive program (Hellqvist 2021). Orem conceptualizes self‐care as a fundamental and purposeful form of behaviour that is primarily focused on leveraging an individual's inherent capabilities and their environmental resources (Tariq 2024). According to Orem, self‐care encompasses the actions individuals take to maintain or enhance their health, well‐being, and life, as well as to prevent or manage illness. The core principle of Orem's model is to empower patients to assume responsibility for their own care, reflecting her belief that people are capable of self‐care (Hellqvist 2021). Within Orem's framework, self‐care is conceptualized as an acquired capability rather than an innate one, and this learned repertoire can address a substantial portion of the requisites arising from illness or health deviation (Fereidooni et al. 2024; Oliveira et al. 2022).

A body of prior research indicates that Orem's self‐care model demonstrates efficacy in alleviating fatigue among patients with COPD (Reiszadeh et al. 2022), multiple sclerosis (Rooddehghan et al. 2023) and breast cancer (Zhang et al. 2024). Furthermore, evidence suggests its potential utility in enhancing the quality of life for elderly populations (Fallah et al. 2026), and individuals suffering from migraine headaches (ZF et al. 2016).

Given that insufficient knowledge and education about lifestyle and self‐care contribute to low awareness among diabetic patients, implementing self‐care management education based on Orem's theory is essential. Therefore, this study was designed to evaluate the impact of Orem's self‐care model on balance and fatigue in patients with diabetic neuropathy, addressing a critical gap in nursing practice and patient‐centred care.

## 2 Methods

### 2.1 Study Design

From 5 January 2023 to 5 May 2023, 70 patients with DPN who were referred to the Shahid Mofateh Clinic, affiliated with Yasuj University of Medical Sciences (YUMS), participated in this randomized clinical trial. The participants were selected via convenience sampling and randomly allocated to the intervention and control groups using block randomization.

### 2.2 Participants

Patients between the ages of 40 and 60 who had been diagnosed with peripheral neuropathy by an internal medicine specialist, based on the Toronto Clinical Scoring System (TCSS), were included in the study.

#### 2.2.1 Inclusion and Exclusion Criteria

##### 2.2.1.1 Inclusion Criteria

1. Willingness to participate in the study.

2. Ability to communicate and respond to questions.

3. Regular use of blood glucose–lowering medications.

4. Presence of DPN symptoms for at least 1 month, such as discomfort and burning pain at rest.

##### 2.2.1.2 Exclusion Criteria

1. Central nervous system disorders, such as multiple sclerosis, Parkinson's disease, or dementia.

2. Skeletal or musculoskeletal deformities of the lower limbs.

3. A history of balance‐impairing Meniere's disease or ear surgery.

4. Diabetic foot ulcers or neurological issues (apart from diabetic neuropathy) that affect gait.

5. The use of immunosuppressive medications (such as corticosteroids) or neuropathic pain relievers (such as paroxetine, gabapentin, pregabalin, or tricyclic antidepressants) within the past 3 months.

### 2.3 Sample Size Determination

The sample size was calculated based on the study by (Naroie et al. 2012), using parameters (*μ*₁ = 3.59, *μ*_2_ = 5.39, *σ*₁ = 2.23, *σ*_2_ = 2.65, *α* = 0.05, 1‐*β* = 0.80, effect size *d* = 0.73) in G*Power software version 3.1.9.7.

An initial sample size of 62 was determined. Considering a 10% dropout rate, the final sample size was increased to 70 participants, with 35 assigned to each group (intervention and control).

### 2.4 Participants

First, 86 people were assessed. However, 16 participants were excluded: 11 did not meet the inclusion criteria, and five declined to participate. In the end, the study included 70 individuals who were enrolled, with 35 in each group (Figure 1).

**FIGURE 1:**
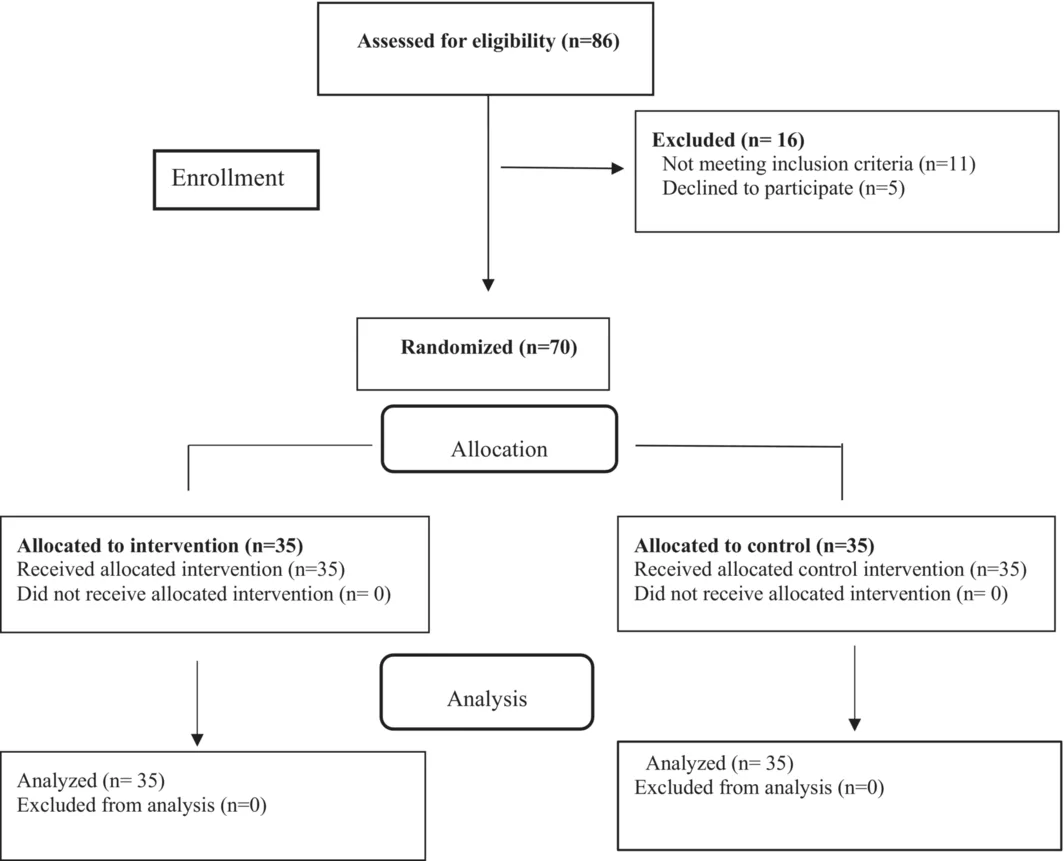
CONSORT flow diagram of participant enrolment, allocation, follow‐up, and analysis.

### 2.5 Randomization

Eligible participants were randomly assigned to the intervention or control group in a 1:1 allocation ratio using block randomization generated through the Sealed Envelope online randomization platform. Considering the total sample size and the two study groups, block sizes of two and four were used. The platform generated a unique identification code and the corresponding group allocation for each participant. Group assignment was carried out by an independent researcher who was not involved in participant recruitment, intervention delivery, data collection, or outcome assessment, according to the computer‐generated randomization sequence.

### 2.6 Blinding

Due to the educational nature of the intervention, blinding of participants was not feasible, and participants were aware of their group allocation throughout the study. However, to minimize the risk of bias, the outcome assessor and the statistician responsible for data analysis were blinded to group allocation. Both remained unaware of participants' assignments until the completion of data collection and statistical analyses.

### 2.7 Intervention Group

The self‐care based on the Orem model (Smith 2019) was tailored to the physical, educational (self‐care), and supportive needs of diabetic neuropathy patients through an Orem needs assessment form. This program was delivered over eight sessions, each lasting 45–60 min (conducted in five groups of seven patients over 2 months in the training room of Shahid Mofateh Educational and Therapeutic Clinic, affiliated with YUMS), by a single trained researcher (a master's student in nursing who had completed the theoretical and practical coursework related to the Orem model). Whenever a patient had a specific self‐care need that was not covered in the group session, a supplementary individual session addressing that need was arranged. This was followed by one‐month self‐care at home using a self‐report checklist. To reduce and prevent complications such as urinary incontinence (Valsalva manoeuvre, pelvic floor muscle exercises, blood sugar control), fatigue (showering, fluid intake, humour and joke‐telling, stress management, engaging activities such as joke‐telling and watching TV, short naps), visual disturbances (periodic examination strategies, dietary control, and use of protective eyewear), muscle cramps and imbalance (warm showers, avoiding cold air and massage, using assistive devices), and pain (humour therapy and distraction techniques), and depression (writing down feelings and humour therapy), the program provided a set of information, behaviours, and performances tailored to the needs of patients with diabetic neuropathy. The self‐care program at home was implemented over the course of 1 month. At the end of this period, the daily self‐report checklists from patients in the intervention group were collected and evaluated. The total duration of the intervention was therefore 3 months (2 months of educational sessions followed by 1 month of home‐based self‐care). The detailed session‐by‐session protocol is provided in Table 1.

**TABLE 1:** Session‐by‐session description of the intervention.

Session	Aim/theme	Content and self‐care strategies taught	Link to Orem's framework
1	Introduction and orientation	Members getting acquainted; defining goals and session rules; explaining how the care model would be implemented	Orientation; educative‐supportive system
2	Understanding the disease	General overview of type 2 diabetes: definition, diagnostic methods, treatments, and complications	Educative‐supportive system
3	Universal self‐care requisites + review	Air, water, food, elimination, activity and rest, solitude and social interaction, prevention of hazards (strategies related to elimination/urinary incontinence integrated here)	Universal self‐care requisites
4	Coping with visual disturbances, muscle cramps, pain, and depression	Vision: periodic examinations, dietary control, protective eyewear. Cramps: warm showers, avoiding cold air, massage. Pain: humour therapy and distraction. Depression: writing down feelings, humour therapy	Health‐deviation self‐care requisites
5	Coping with fatigue (part 1) + review	Daily‐activity planning, home/work environment design, assistive blood‐glucose devices, bathing, nutrition, salt restriction, humour therapy, stress avoidance, pain relief, light exercise, yoga relaxation, acupuncture	Health‐deviation self‐care requisites
6	Coping with fatigue (part 2)	Showering, fluid intake, humour and joke‐telling, stress management, engaging activities (joke‐telling, watching TV), short naps	Health‐deviation self‐care requisites
7	Improving body balance	Using a walker/cane, home environment design, wearing flat or low‐heeled shoes, walking with feet slightly apart, looking at the feet while walking	Health‐deviation requisites; prevention of hazards
8	Consolidation and self‐report training	Addressing remaining questions; training patients to complete the self‐care self‐report checklist for the 1‐month home period	Transition to independent self‐care

### 2.8 Control Group

Throughout the study period, the control group received only routine care. At the end of the study, for ethical reasons, the control group received the self‐care program in a single group session, along with an educational booklet, and their questions were answered.

### 2.9 Data Collection

Demographic data collected included sex, marital status, educational attainment, and underlying medical conditions. Berg Balance Scale Questionnaire.

#### 2.9.1 The Berg Balance Scale, Developed by Katherine Berg, Assesses Static and Dynamic Balance in Neurological Patients

It consists of 14 balance tests, each scored from zero to four. A total score of 20 or less indicates that the patient requires a wheelchair; a score of more than 20 but less than 40 indicates that the patient needs assistance when walking; and patients with scores above 40 can walk independently (Joa 2024). The validity and reliability of this questionnaire (94% and 90%, respectively) have been confirmed in the Iranian population (Sohrabi et al. 2020).

#### 2.9.2 Multidimensional Fatigue Questionnaire

This questionnaire was developed by Smets et al. (1995) and consists of 20 items across five subscales: general fatigue (4 items), physical fatigue (4 items), reduced activity (4 items), reduced motivation (4 items), and mental fatigue (4 items). The questionnaire uses a 5‐point Likert scale for scoring (from 1 = “completely true” to 5 = “completely false”), with higher scores indicating greater fatigue (Smets et al. 1995). Hafezi et al. (2010) validated its use in Iran, reporting reliability and validity values of 0.93 and 0.85, respectively (Hafezi et al. 2010).

#### 2.9.3 Orem's Health Model Assessment and Recognition Form the Orem's Health Model Assessment and Recognition Form Consists of Two Sections

1. Self‐care status assessment: (A) Changes in self‐care ability, (B) self‐care ability, and (C) dependent self‐care ability.
2. Self‐care needs assessment: (A) Universal needs, (B) developmental needs, and (C) health deviation needs.

### 2.10 Data Analysis

Data were analysed using SPSS software (version 27.0). Continuous variables are presented as mean ± standard deviation (SD), and categorical variables as frequency (percentage). Baseline demographic characteristics were compared using the independent‐samples *t*‐test for continuous variables and the chi‐square test for categorical variables. For continuous outcomes, analysis of covariance (ANCOVA) was used to compare post‐intervention scores between groups while adjusting for baseline scores. For balance, the group × baseline balance interaction was examined, and adjusted between‐group differences were estimated at low (−1 SD), mean, and high (+1 SD) levels of baseline balance. Adjusted means, adjusted mean differences with 95% confidence intervals (CIs), *F* statistics, *p* values, and partial eta squared (*η* ^2^ *p*) were reported. A *p* < 0.05 was considered statistically significant.

## 3 Results

Eighty‐six diabetic neuropathy patients were assessed in this study. Sixteen patients were excluded from the study (11 were not eligible, and five declined to participate).

Ultimately, 70 diabetic neuropathy patients participated, with 35 in the intervention group and 35 in the control group (Figure 1). The participants' mean age (standard deviation) was 54.88 (7.49) years, ranging from 40 to 65 years. Table 2 displays the demographic details of the individuals in both groups, including sex, marital status, education level, and underlying illness.

**TABLE 2:** Comparison of the demographic characteristics of the study groups.

Variable		Intervention	Control	*p*
Age (years) (mean ± SD)		53.88 ± 7.17	55.88 ± 7.78	0.26^t^
Sex *N* (%)	Male	15 (53.60)	13 (46.40)	0.80^χ2^
	Female	20 (47.60)	22 (52.44)	
Educational status *N* (%)	< Diploma Diploma	34 (49.30)	35 (50.70)	1.00^χ2^
	> Diploma	1 (100.00)	0 (00.00)	
Marital status *N* (%)	Single	4 (40.00)	6 (60.00)	0.73^χ2^
	Married	31 (51.70)	29 (48.30)	
Underlying disease *N* (%)	Yes	22 (48.90)	23 (51.10)	1.00^χ2^
	No	13 (52.00)	12 (48.00)	

^t^ Independent test.

^χ2^ Chi‐square test.

Table 2 indicates that there were no significant differences between the intervention and control groups with respect to background variables (age, sex, marital status, education level, and underlying disease) (*p* > 0.05).

Table 3 presents the ANCOVA results for post‐intervention fatigue scores in the intervention and control groups.

**TABLE 3:** Comparison of post‐intervention fatigue scores between the intervention and control groups using ANCOVA.

Outcome	Group	Pre‐test mean ± SD	Post‐test mean ± SD	Adjusted post‐test mean (95% CI)	Adjusted mean difference (95% CI)	F(1,67)	*p*	Partial *η* ^2^
Fatigue	Intervention	56.00 ± 7.90	46.77 ± 8.09	45.40 (44.22–46.58)	−9.40 (−11.09 to −7.71)	123.05	< 0.001	0.647
	Control	52.97 ± 6.92	53.43 ± 6.97	54.80 (53.62–55.98)	_			

After adjustment for baseline fatigue scores, the adjusted mean fatigue score was 45.40 (95% CI: 44.22–46.58) in the intervention group and 54.80 (95% CI: 53.62–55.98) in the control group. The adjusted mean difference between groups was −9.40 points (95% CI: −11.09 to −7.71) and was statistically significant (F(1, 67) = 123.05, *p* < 0.001). The effect size was partial *η* ^2^ = 0.647. These findings indicate that, after controlling for baseline fatigue, the intervention was associated with a significant reduction in fatigue compared with the control group.

The unadjusted pre‐ and post‐intervention mean fatigue scores are presented in Figure 2.

**FIGURE 2:**
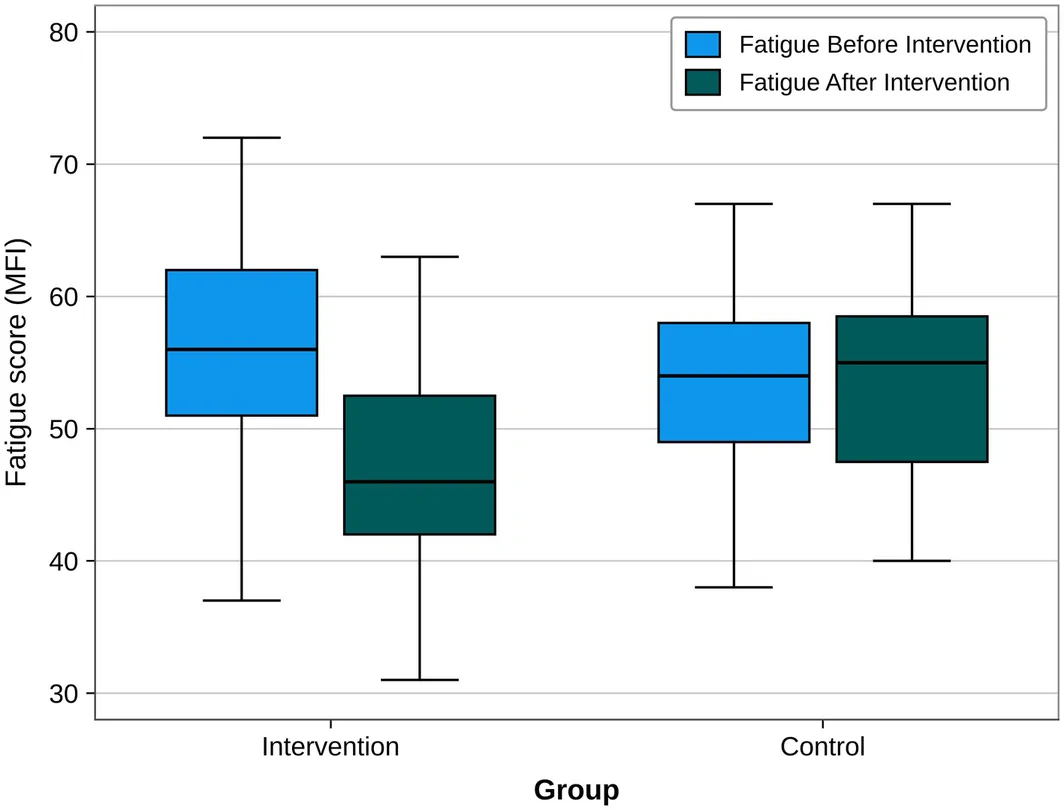
Unadjusted pre‐ and post‐intervention mean fatigue scores in the intervention and control groups.

Table 4 presents the ANCOVA results for post‐intervention balance scores, adjusted for baseline balance, including the group × baseline balance interaction.

**TABLE 4:** ANCOVA of post‐intervention balance scores by treatment group and baseline balance.

Baseline balance	Intervention adjusted mean (95% CI)	Control adjusted mean (95% CI)	Adjusted mean difference (95% CI)	F(1,66)	*p*	Partial *η* ^2^
−1 SD (27.42)	34.90 (33.72–36.08)	27.89 (27.01–28.76)	**7.01 (5.54–8.48)**	90.57	< 0.001	0.578
Mean (35.74)	41.33 (40.63–42.04)	35.56 (34.86–36.26)	**5.77 (4.77–6.76)**	134.41	< 0.001	0.671
+1 SD (44.07)	47.76 (46.76–48.76)	43.24 (42.24–44.23)	**4.52 (3.11–5.93)**	41.05	< 0.001	0.383

*Note:* The bold values indicate statistically significant differences (*p* < 0.05) between the intervention and control groups.

ANCOVA showed a significant group × baseline balance interaction (*F*(1,66) = 5.687, *p* = 0.020, partial *η* ^2^ = 0.079), indicating that the between‐group difference in post‐intervention balance scores varied according to baseline balance. At one standard deviation below the mean baseline balance (27.42), the adjusted mean balance score was 34.90 (95% CI: 33.72–36.08) in the intervention group and 27.89 (95% CI: 27.01–28.76) in the control group, with an adjusted mean difference of 7.01 points (95% CI: 5.54–8.48; *F*(1,66) = 90.57, *p* < 0.001). At the mean baseline balance score (35.74), the corresponding adjusted means were 41.33 (95% CI: 40.63–42.04) and 35.56 (95% CI: 34.86–36.26), respectively, with a between‐group difference of 5.77 points (95% CI: 4.77–6.76; *F*(1,66) = 134.41, *p* < 0.001). At one standard deviation above the mean baseline balance (44.07), the adjusted means were 47.76 (95% CI: 46.76–48.76) and 43.24 (95% CI: 42.24–44.23), respectively, with a difference of 4.52 points (95% CI: 3.11–5.93; *F*(1,66) = 41.05, *p* < 0.001). Thus, ANCOVA indicated significantly higher post‐intervention balance scores in the intervention group than in the control group across all three levels of baseline balance, while the magnitude of the between‐group difference decreased as baseline balance increased. Unadjusted pre‐ and post‐intervention mean balance scores are presented in Figure 3.

**FIGURE 3:**
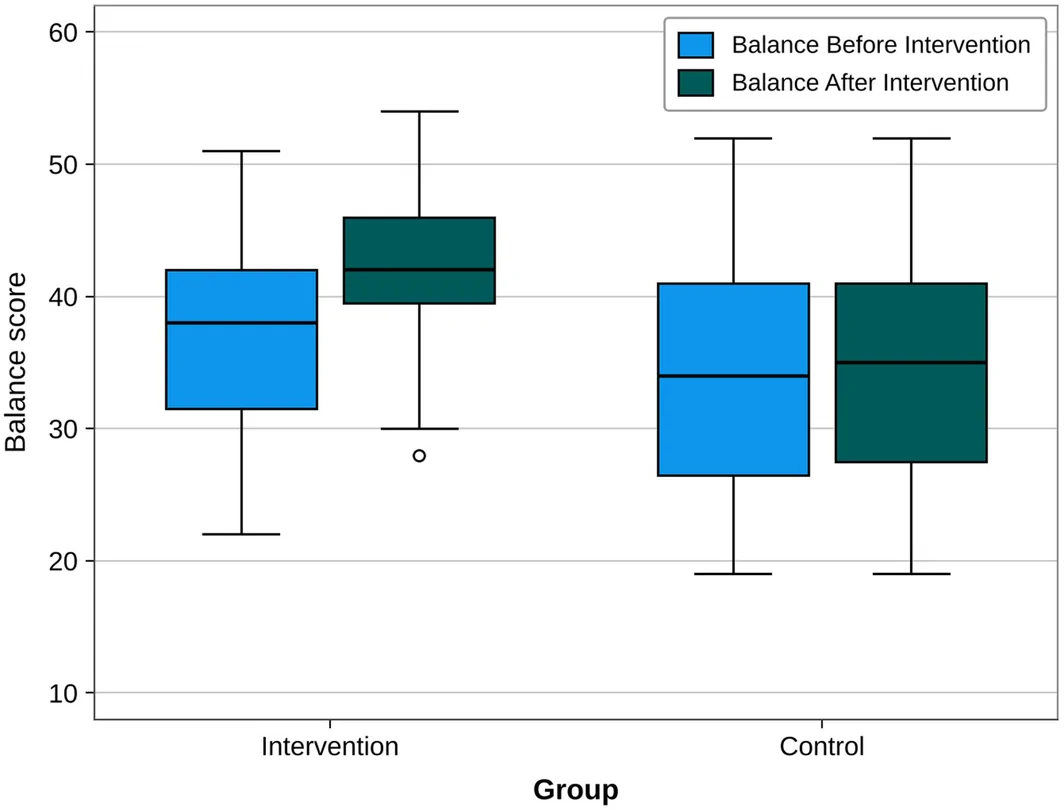
Unadjusted pre‐ and post‐intervention mean balance scores in the intervention and control groups.

## 4 Discussion

This randomized clinical trial examined the effects of Orem's self‐care model on balance and fatigue in patients with diabetic neuropathy. The principal findings were that the intervention was beneficial for both outcomes after accounting for their baseline values. The balance response also varied according to initial balance performance, with greater benefit among patients who had poorer balance at baseline. These findings suggest that an Orem‐based approach may be particularly relevant for patients with greater functional limitations, while also supporting its potential value for fatigue management.

The improvement in balance is consistent with previous exercise‐based studies in patients with DPN. Combined programs incorporating range‐of‐motion, strengthening, balance, and gait exercises have improved balance and reduced fall risk, while trampoline‐based exercise has been associated with better static and dynamic balance (Farhan et al. 2019; Rafiei et al. 2022). Evidence from older populations also indicates that structured physical exercise can improve static and dynamic balance (Papalia et al. 2020), although differences in age and clinical characteristics limit direct comparison with patients with diabetic neuropathy. Venkataraman et al. similarly found that strength and balance training improved functional performance and balance confidence in individuals with DPN, but did not improve overall health‐related quality of life (Abiodun et al. 2024). Accordingly, the improvement in balance observed in the present study should not be assumed to translate directly into fewer falls or better quality of life, as neither outcome was measured. The variation in intervention response according to baseline balance provides additional clinical insight. Patients with poorer initial balance may have had greater capacity for measurable functional improvement and may have derived more benefit from the balance‐related elements of the program. Conversely, participants with better baseline performance had less scope for improvement. This pattern supports assessing initial balance when planning and individualizing self‐care interventions. However, baseline balance was analysed as a continuous characteristic rather than as three distinct patient subgroups, and the observed effect modification should be confirmed in adequately powered studies.

The reduction in fatigue is also clinically relevant because fatigue in diabetes is multifactorial and may arise from interacting glycaemic, medical, psychological, nutritional, and lifestyle factors (Venkataraman et al. 2019). Fatigue may, in turn, reduce patients' capacity to engage consistently in disease management, creating a cycle of low energy and inadequate self‐care. An Orem‐based program may interrupt this cycle by strengthening patients' knowledge, self‐care agency, problem‐solving abilities, and participation in daily disease management. Nevertheless, these mechanisms were not measured directly and should therefore be regarded as plausible explanations rather than established mediators.

The direction of the fatigue finding is consistent with evidence from other chronic conditions. Education designed to support fatigue management and self‐care reduced fatigue and improved self‐care agency among patients receiving haemodialysis (Kalra and Sahay 2018). Orem‐based interventions have also reduced fatigue among patients with COPD and multiple sclerosis (Deveci and Aydın 2022; Reiszadeh et al. 2022) Improvements in quality of life and self‐care behaviours have been reported in patients with hypothyroid goitre and diabetic foot ulcers, respectively (Rooddehghan et al. 2023; Shosha et al. 2024). Although these studies support the broader applicability of Orem's model, differences in disease processes, intervention content, and outcome measurement mean that their findings cannot be directly extrapolated to diabetic neuropathy. The present findings extend this evidence by supporting the potential value of a theoretically guided self‐care approach for addressing functional and symptom‐related needs in patients with diabetic neuropathy. From a nursing perspective, incorporating individualized self‐care assessment and Orem‐based interventions into routine care may strengthen evidence‐based, patient‐centred management of fatigue and balance problems.

The present findings also reinforce the nursing role in identifying self‐care deficits and providing individualized supportive–educative care. Diabetes self‐care practices, including physical activity, healthy eating, blood glucose monitoring, medication adherence, and foot care, have been associated with better glycaemic control and fewer complications (Venkataraman et al. 2019). However, evidence regarding education alone is more cautious: patient education may improve foot‐care knowledge and self‐reported behaviour in the short term, but evidence that it prevents ulcers or amputations remains insufficient (Alshammari et al. 2023). Knowledge, diabetes‐related distress, education, and family support have been identified as predictors of foot self‐care (Sari et al. 2020). Inadequate knowledge remains an important barrier (Alshammari et al. 2023).

Coaching interventions may also improve patients' knowledge of strategies intended to prevent deterioration of DPN (Mohamed et al. 2024). These findings support integrating structured self‐care education into nursing care while tailoring its content to patients' functional status, fatigue, knowledge, psychological burden, and available family support. Improved self‐care engagement may explain part of the observed benefit; however, self‐care behaviour was not evaluated as a mediator in the present study. By encouraging active participation in self‐care and disease‐management activities, the intervention may support patients' active participation in self‐management and confidence in managing diabetes‐related symptoms. From a nursing perspective, this approach can strengthen nurse‐led chronic disease management through individualized assessment, education, supportive care, and ongoing follow‐up (Nasiri et al. 2023).

In practice, nurses in outpatient diabetes clinics, primary care, and community settings can integrate this approach into routine care through regular assessment of self‐care needs, fatigue, and balance, followed by individualized education, supportive interventions, and ongoing follow‐up. Such integration may facilitate continuity of care and support individualized management of patients with diabetic neuropathy.

## 5 Limitations

Program of additional contact and support. Although the analyses controlled for baseline outcomes, HbA1c and diabetes duration were unavailable; therefore, despite random allocation, imbalance in unmeasured prognostic factors cannot be excluded. Participants could not be blinded, creating potential performance and expectancy biases. Blinding of the outcome assessor and statistician reduced detection and analytical bias, but self‐reported fatigue remained susceptible to reporting bias. Outcomes were assessed only at the end of the three‐month study period, so their durability is unknown. In addition, the single‐centre convenience sample and relatively small sample size limit generalisability and warrant caution when interpreting the interaction between treatment group and baseline balance. Falls, quality of life, self‐care behaviour, intervention mediators, and the clinical importance of the observed score differences were not evaluated.

Future multicentre trials should include larger and more diverse samples, attention‐matched controls, longer follow‐up, and measures of falls, quality of life, glycaemic, adherence, and self‐care behaviour. Prespecified analyses should examine whether baseline balance modifies the intervention effect. Factorial or dismantling designs could identify the most effective components, while qualitative studies could clarify how fatigue, motivation, family support, and digital support influence self‐care engagement.

## 6 Conclusion

An Orem‐based self‐care program improved balance and reduced fatigue in patients with diabetic neuropathy over the study period. The greater balance benefit among patients with poorer initial balance suggests that baseline functional status may help identify those most likely to benefit. These findings support the potential integration of individualized Orem‐based self‐care education into nursing care for diabetic neuropathy. Larger multicenter trials with active control conditions and longer follow‐up are required to confirm the clinical importance, mechanisms, and durability of these effects.

## Author Contributions

**Atefeh Sadeghi:** conceptualization, investigation, writing – original draft, writing – review and editing, data curation, resources, software. **Mohammad Malekzadeh:** methodology, investigation, writing – original draft, formal analysis, software, validation. **Abolfazl Dehbanizadeh:** investigation, writing – original draft, writing – review and editing, resources. **Seyed Javad Sadat:** conceptualization, supervision, investigation, writing – original draft, writing – review and editing, methodology, resources, project administration.

## Funding

The authors have nothing to report.

## Ethics Statement

This study was registered with the Iranian Registry of Clinical Trials (code: IRCT20221016056186N1) and approved by the Yasuj University of Medical Sciences Research Ethics Committee (code: IR.YUMS.REC.1401.105). Following an explanation of the study's objectives, each participant provided written informed consent. The right to withdraw at any time and the confidentiality of the data were guaranteed, and participation was entirely optional. The study adhered to the principles of the Declaration of Helsinki, ensuring respect for autonomy, privacy, and welfare of all participants. Before enrollment, all participants received detailed information regarding the study objectives, procedures, potential benefits and risks, and their right to withdraw from participation at any time without penalty.

## Consent

The authors have nothing to report.

## Conflicts of Interest

The authors declare no conflicts of interest.

## Data Availability

The data that support the findings of this study are available on request from the corresponding author. The data are not publicly available due to privacy or ethical restrictions.
